# Supplementary material for: Paediatric Deep Neck Infection—The Risk of Needing Intensive Care
Source: Children (Basel). 2022 Jun 29;9(7):979. doi: 10.3390/children9070979 (PMC9315740; doi:10.3390/children9070979)
Supplement: Supplementary file 1 [file children-09-00979-s001.zip › children-1754042-supplementary.pdf]

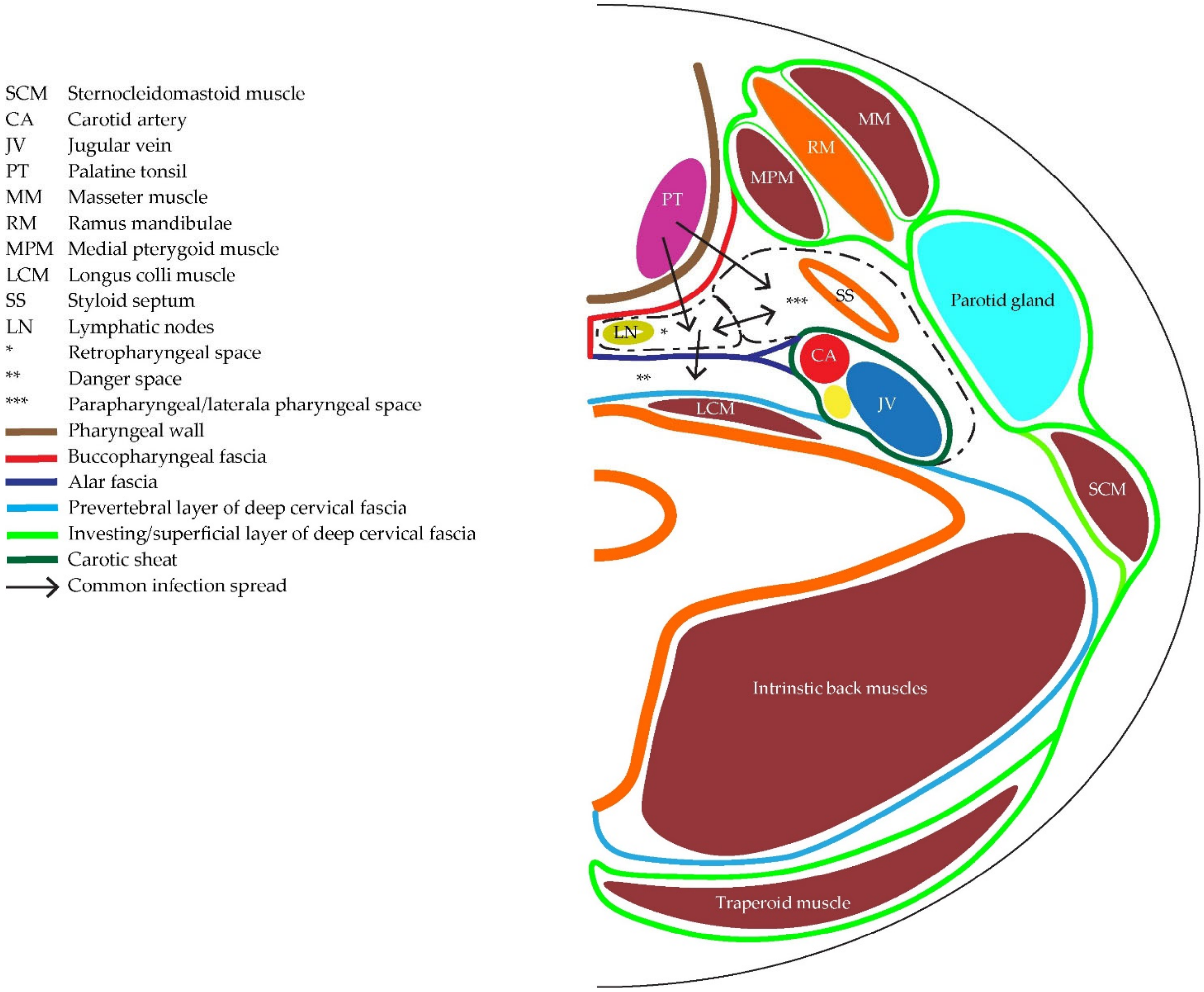

Figure S1.

Title: Schematic diagram of deep neck fascia and spaces

Caption: This simplified diagram of the deep neck fascia and spaces shows mainly the relationship among in children most frequently involved spaces. It is not anatomically accurate, particularly it does not represent specific transversal plane.
